# Insight into the role of grafting and arbuscular mycorrhiza on cadmium stress tolerance in tomato

**DOI:** 10.3389/fpls.2015.00477

**Published:** 2015-06-26

**Authors:** Pradeep Kumar, Luigi Lucini, Youssef Rouphael, Mariateresa Cardarelli, Raviraj M. Kalunke, Giuseppe Colla

**Affiliations:** ^1^Indian Council of Agricultural Research–Central Arid Zone Research Institute, JodhpurIndia; ^2^Department of Agriculture, Forestry, Nature and Energy, University of Tuscia, ViterboItaly; ^3^Institute of Environmental and Agricultural Chemistry, Università Cattolica del Sacro Cuore, PiacenzaItaly; ^4^Department of Agricultural Sciences, University of Naples Federico II, PorticiItaly; ^5^Consiglio per la Ricerca in Agricoltura e l’Analisi dell’Economia Agraria, Centro di Ricerca per lo Studio delle Relazioni tra Pianta e Suolo, RomaItaly

**Keywords:** antioxidant enzymes, cadmium, gene expression, metabolomic, rootstock, *Solanum lycopersicum* L.

## Abstract

Physiological, biochemical, metabolite changes, and gene expression analysis of greenhouse tomato (*Solanum lycopersicum* L.) were investigated in two grafting combinations (self-grafted ‘Ikram’ and ‘Ikram’ grafted onto interspecific hybrid rootstock `Maxifort'), with and without arbuscular mycorrhizal (AM), exposed to 0 and 25 μM Cd. Tomato plants responded to moderate Cadmium (Cd) concentration by decreasing yield and crop growth parameters due to the accumulation of Cd in leaf tissue, inhibition of the PS II activity, reduced nutrients translocation, and also to the oxidative stress as evidenced by enhanced hydrogen peroxide (H_2_O_2_) generation, ion leakage, and lipid peroxidation. AM inoculation significantly enhanced the metal concentration in shoots and reduced growth and yield. The Ikram/Maxifort combination induced higher antioxidant enzymes, higher accumulation of proline and reduction of lipid peroxidation products. This suggests that the use of Maxifort rootstock in tomato has a high reactive oxygen species scavenging activity since lower H_2_O_2_ concentrations were observed in the presence of Cd. The higher crop performance of Ikram/Maxifort in comparison to Ikram/Ikram combination was also due to the improved nutritional status (higher P, K, Ca, Fe, Mn, and Zn) and increased availability of metabolites involved in cadmium tolerance (phytochelatin PC2, fructans, and inulins). The up-regulation of *LeNRAMP3* gene in leaf of Ikram/Maxifort could explain the better nutritional status of interspecific grafting combination (higher Fe, Mn, and Zn).

## Introduction

Cadmium (Cd) is the most hazardous heavy metal element for plant growth with high toxicity even at low level (5–10 μg g^-1^dry weight; Dong et al., 2006; White and Brown, 2010). Cd contamination in agricultural soils may come from various sources, including the use of phosphate fertilizers, pesticides, organic manures, and sewage sludge (Dong et al., 2007; Uraguchi et al., 2009). Owing to its high solubility and mobility, Cd is readily absorbed by plant roots and translocated to the shoots (Dong et al., 2007; Gallego et al., 2012) and even to the fruits through phloem mediated transport (Hèdiji et al., 2010; Bertoli et al., 2012) thereby, endangering not only plant productivity, but also human health via the food chain (Dong et al., 2007). Cd is a non-essential element for plants and is taken up by plants through transporters of certain essential elements due to their low metal specificity and/or through Ca transport mechanism (Gallego et al., 2012).

In vegetable crops, Cd stressful conditions have been reported to disturb physiological, biochemical, and metabolic processes leading to growth inhibition (López-Millán et al., 2009; Hèdiji et al., 2010; Sharma et al., 2010). Particularly in tomato plants, excess Cd causes inhibition of chlorophyll and carotenoids biosynthesis and substantial inhibition of the photosystem (PS) II activity. Further, it causes significant alteration in nutrient uptake and translocation of K, Ca, Mg, Mn, Fe, and Zn (Djebali et al., 2005; Dong et al., 2006; Hèdiji et al., 2010; Bertoli et al., 2012). Therefore, Cd-exposed plants show visible symptoms such as reduction of shoot and root biomass and leaf chlorosis and necrosis (Smeets et al., 2005; Shahabivand et al., 2012). At low Cd concentrations in soil or water, these visible symptoms are less marked, but various cellular processes may still be affected (Smeets et al., 2005; Gratão et al., 2008; Hèdiji et al., 2010). At cell level, Cd has been demonstrated to induce oxidative stress in plants by producing reactive oxygen species (ROS) that disrupt cellular homeostasis and damage proteins, pigments, lipid peroxidation, and ion leakage, which in turn leads to membrane damage and enzymes inactivation (Sharma and Dubey, 2005; Pitzschke et al., 2006; Gratão et al., 2008). In order to cope with such toxic effects derived from ROS, plants may employ integrated antioxidant defense mechanisms (Gallego et al., 2012). The antioxidant defense system comprises antioxidant enzymes such as superoxide dismutase (SOD), catalase (CAT), ascorbate peroxidase (APX), and guaiacol peroxidase (GPX), as well as non-enzymatic antioxidants such as reduced glutathione (GSH), ascorbic acid (AsA), α-tocopherol and carotenoids (Gratão et al., 2008, 2012; Gill and Tuteja, 2010; Gallego et al., 2012). Moreover, plants exposed to toxic heavy metals have also been shown to regulate some metabolites to cope with the toxic effects of Cd. These include, among others, proline, ascorbate, carotenoids, glucosinolates, and phytochelatinsas well as phytohormones.

Numerous attempts have been made to enhance heavy metal tolerance of vegetable crops by traditional breeding and genetic engineering with limited success (Savvas et al., 2010). Consequently, to alleviate the negative effects of Cd-toxicity, various strategies have been suggested. One of these is a grafting strategy involving rootstocks that are known to enhance plants’ ability in mitigating the adverse environmental conditions and it can be a faster and sustainable tool (Colla et al., 2010; Savvas et al., 2010; Schwarz et al., 2010). Evidence of exploiting grafting against heavy metal in vegetables came to light only a few years ago from the study done by Takeda et al. (2007). The authors demonstrated that grafting eggplant onto *Solanum torvum* was effective in reducing Cd concentrations in the eggplant fruit by up to 25% when compared to those of ungrafted plants. This phenomenon was further studied and reported to be attributed by either reduced Cd flux or lower xylem loading in *S. torvum* (Arao et al., 2008; Mori et al., 2009; Yamaguchi et al., 2011). In cucumber, Savvas et al. (2013) demonstrated that the pumpkin rootstock ‘Power’ could efficiently restrict Cd concentration in plant tissues by 12–50% in comparison with other grafting combinations. The proposed mechanism for reduced aerial Cd contents was found to be related to the higher exclusion efficiency of ‘Power’ rootstock for Cd at the plasma membrane of root cells.

Another promising and environment friendly tool to overcome Cd toxicity in contaminated soils would be through inoculation with beneficial microorganisms such as arbuscular mycorrhizal (AM) fungi. AM are beneficial associations between soil fungi and plant roots which is found in 83% of higher plants (Smith and Read, 2008). The association of AM fungi with plant roots can enhance plant growth and resistance to stressful edaphic conditions including toxicity produced by heavy metals (Ouziad et al., 2005; Shahabivand et al., 2012). Cd-induced growth depression was highly mitigated by mycorrhiza [*Rhizophagus* (formely named *Glomus*) *intraradices* or *Funneliformis* (formely named *Glomus*) *mosseae*] in two pea (*Pisum sativum* L.) genotypes and *Medicago truncatula* (Repetto et al., 2003, 2007; Aloui et al., 2012). The former authors demonstrated that the mycorrhizal process may modulate the expression of proteins involved in Cd-responses in *P. sativum* and *M. truncatula*. Furthermore, the effects of AM fungi on heavy metal accumulation has been reported in several studies, but with contrasting results. Lin et al. (2007) observed that colonization by *F. mosseae* decreased the concentration of metals in the shoots of three leguminous plants thereby reducing the heavy metal toxicity. Contrarily other studies demonstrated no change (Wu et al., 2007a) or even an increase in the uptake and accumulation of heavy metals in mycorrhizal (*F. mosseae* or *R. intraradices*) tissue plants (Joner and Leyval, 2001; Lingua et al., 2008). It is worth noting that the translocation and accumulation of heavy metals may depend on fungus–plant interactions, levels, and types of metal (Leyval et al., 1995; Gamalero et al., 2009; Rouphael et al., 2015).

There is scarce or null information about the influence of grafting and AM fungi on physiological and metabolic changes under heavy metal stress in vegetable crops such as tomato. Our hypothesis was that grafting tomato onto vigorous rootstock with or without AM inoculation, may raise Cd tolerance through limiting heavy metal uptake by roots and/or its translocation to the shoots, or by facilitating metal detoxification process by regulating the level of antioxidants and of certain metabolite concentrations in plants.

The aim of this study was to investigate the effects of grafting and inoculation with the AM fungus *R. irregularis*, alone or in combination, on physiological, biochemical, metabolite, and gene expression changes of greenhouse tomato plant under moderate external Cd concentration, which is common in current agriculture practice as stated by López-Millán et al. (2009) and Uraguchi et al. (2009).

## Materials and Methods

### Growth Conditions and Treatments

Tomato (*Solanum lycopersicum* L. cv. Ikram, Syngenta, Milan, Italy) plants self-grafted, or grafted onto a vigorous interspecific hybrid rootstock Maxifort (*S. lycopersicum* × *S. habrochaites*, De Ruiter/Monsanto, Bergschenhoek, The Netherlands) were produced using the splice graft technique. During the culture, plants were grown in a greenhouse from April 24 to August 25, 2014. At four true leaf stage tomato seedlings were transferred into pots (6 L) containing quartziferous sand. Pots were arranged in double rows in separate plastic covered channel providing sufficient slope to enable drainage collection into tanks fixed at the lateral end of each channel. The plant density was 3.7 plants m^-2^. Pollination was facilitated by bumble bees.

The experiment was designed as a factorial combination of two Cd concentrations [0 (control), or 25 μM], two grafting combinations (self-grafted Ikram/Ikram, or Ikram/Maxifort), and two mycorrhizal treatments (with AM, +AM or without AM, −AM). The treatments were arranged in a randomized complete-block design with four replicates per treatment. Each experimental unit consisted of three plants. Prior to transplanting, half of the transplants received a mycorrhizal inoculum carrying 50 spores g^-1^ of *R. irregularis*. Inoculum was applied in the transplanting hole at a rate of 5 g plant^-1^.

Plants were fed with a basic nutrient solution containing: 14.0 mM N–NO_3_, 1.6 mM S, 0.3 mM P, 6.0 mM K, 4.5 mM Ca, 1.5 mM Mg, 20 μM Fe, 9 μM Mn, 0.3 μM Cu, 1.6 μM Zn, 20 μM B, and 0.3 μM Mo. After an initial growth period of 28 (May 21), Cd was added as chloride to the basic nutrient solution at a concentration of 25 μM. The pH of the nutrient solution for all treatments was 5.6 ± 0.2. Deionized water was used for the preparation of all nutrient solutions. Nutrient solution was pumped from independent supply tanks through a drip irrigation system, with one emitter per plant of 2 L h^-1^ flow rate (Rouphael et al., 2008). The duration of each irrigation event was tuned to provide at least 35% of the nutrient solution draining from the pots (Rouphael et al., 2004; Rouphael and Colla, 2005).

### Plant Biomass

Fruits were harvested from July 2 until August 22 and marketable fruits were weighed. At final plant harvest (August 25, 124 days after transplanting, DAT), morphological parameters were recorded. Each plant was divided in three fractions, i.e., leaves, stems, and roots. Fresh and dry weights of each fraction and the root to shoot ratios were determined. Shoot biomass was equal to the sum of aerial vegetative plant parts (leaves + stems). Leaf area was measured with Li-3000 area meter (Li-CorDelta-T Devices Ltd., Cambridge, UK).

For the biochemical and enzyme assays, fresh leaf samples from the third leaves from top were harvested from each plant and immediately frozen in liquid nitrogen and stored at −80°C for later antioxidant enzyme activity analysis.

### Root Colonization

A small fraction of the root system was carefully washed in tap water, cut into 1 cm root pieces. Root samples were cleared with10% KOH, stained with 0.05% trypan blue in lactophenol as described by Phillips and Hayman (1970), and microscopically examined for AM fungi colonization by determining the percentage of root segments containing arbuscules + vesicles using a gridline intercept method (Giovannetti and Mosse, 1980).

### Chlorophyll Fluorescence Measurements

The chlorophyll fluorescence was recorded on 20 min dark-adapted leaves (in third–fourth from top; three measurements per plant) by means of a chlorophyll fluorometer Handy PEA (Hansatech Instruments Ltd, King’s Lynn, UK) with an excitation source intensity higher than 3000 μmolm^-2^s^-1^ at the sample surface. The minimal fluorescence intensity (F_0_) in a dark-adapted state was measured in the presence of a background weak light signal (about 2–3 μmol photons m^-2^s^-1^). The maximal fluorescence level in the dark-adapted state (F_m_) was induced by 0.8 s saturating light pulse (3000 μmol photons m^-2^s^-1^). The maximum quantum yield of open PSII (F_v_/F_m_) was calculated as (F_m_-F_0_)/F_m_, as described by Arena et al. (2005).

### Chlorophyll and Carotenoids Determination

Total chlorophyll and carotenoid (mg g^-1^ FW)were extracted by homogenization of fresh leaf tissues (0.5 g) in acetone (80%). The resulting extracts were centrifuged at 4,800*g* for 20 min. Chlorophyll *a*, chlorophyll *b*, and carotenoid contents were determined by taking absorbance of the supernatant at 470, 647, and 664 nm by a UV–Vis spectrophotometer (Perkin Elmer, Norwalk, CT, USA) following the method of Lichtenhaler and Wellburn (1983).

### Lipid Peroxidation and Electrolyte Leakage Determination

The Cd-induced oxidative damage (membrane lipid peroxidation) in fresh leaf tissue was estimated by measuring the malondialdehyde (MDA) concentrations. Leaf tissues were homogenized in 0.1% (w/v) trichloroacetic acid (TCA) solution in 1:3 ratio. After centrifugation (15 min, 12,000 *g* at 4°C), an aliquot of the supernatant was added to 0.5% thiobarbituric acid (TBA) made in 20% TCA and heated at 95°C for 30 min. After rapid cooling on ice, the mixture was centrifuged at 10,000 *g* for 10 min. The absorbance was recorded at 532 and 600 nm using UV-Vis spectrophotometer. The MDA concentration was calculated from the difference between the absorbance values at 532 and 600 nm (Ben Amor et al., 2005).

The electrolyte ion leakage (EL) was determined as described by Lutts et al. (1995). Ten randomly chosen mature leaves per experimental unit were taken and cut into 1-cm segments. Leaf samples were placed in individual stoppered vials containing 10 mL of distilled water. The samples were incubated at room temperature (25°C) on a shaker (100 rpm) for 24 h. Electrical conductivity of the bathing solution (EC_1_) was read after incubation. The same samples were then placed in an autoclave at 120°C for 20 min and a second reading of the EC (EC_2_) was made after cooling the solution to room temperature. The EL was calculated as EC_1_/EC_2_ and expressed as percentage.

### Hydrogen Peroxide Content

Hydrogen peroxide content was determined according to Sergiev et al. (1997). Tissue powder was homogenized in ice bath by adding 0.1% (W/V) TCA in 1:3 ratio (W/V). After centrifugation (12,000 *g* for 30 min) of the homogenate, supernatant (0.5 mL) was added to 0.5 mL 10 mM K-phosphate buffer (pH 7.0) and 1 mL 1 M potassium iodide. The absorbance was measured at 390 nm. H_2_O_2_ was used as a standard and the content was expressed as μmol H_2_O_2_g^-1^ fresh wt. tissue.

### Antioxidant Enzyme Analyses

Frozen leaf tissues were homogenized with three volumes of an ice-cold extraction buffer (0.05 M potassium phosphate buffer, pH 7.0) containing 0.1% (w/v) AsA, 1% (w/v) polyvinyl polypirrolidone, 1 mM Na_2_-EDTA, and 0.1% (v/v) Triton X-100. After centrifugation (15,000*g*, 30 min, 4°C) the supernatant was set aside for the determination of the enzymes activity by a spectrophotometer (Perkin Elmer, Norwalk, CT, USA). CAT (EC 1.11.1.6) activity was measured according to Havir and McHale (1989). Enzyme activity was evaluated by following the decomposition of H_2_O_2_ at 240 nm for 1 min and calculated using the extinction coefficient (0.036 mM^-1^ cm^-1^). Activity of APX (EC 1.11.1.11) was measured following the decrease of absorbance at 290 nm for 1 min (Nakano and Asada, 1981) corresponding to the oxidation of AsA. APX activity was calculated using its extinction coefficient (2.8 mM^-1^ cm^-1^). Activity of GPX (EC 1.11.1.7) was measured according to with Chance and Maehly (1955). Enzyme activity was evaluated following the increase of absorbance at 470 nm for 40 s due to guaiacol oxidation and calculated using the extinction coefficient (26.6 mM^-1^ cm^-1^). The specific enzyme activity for all enzymes was expressed as mmol mg^-1^ protein min^-1^.

### Proline Analysis

Proline content (mg g^-1^ FW) was determined as described by Bates et al. (1973). Leaf tissue (0.5 g) was homogenized in 10 mL of 30 g L^-1^sulfosalicylic acid and the homogenate was filtered through Whatman No. 2 filter paper. Then 2 mL of filtrate was reacted with 2 mL of acid-ninhydrin (1.25 g of ninhydrin in 30 mL of glacial acetic acid and 20 mL of 6 mol L^-1^ phosphoric acid) and 2 mL of glacial acetic acid in a test tube at 100°C for 1 h. The reaction was terminated in an ice bath and then 4 mL of toluene was added and the product of the reaction was extracted by vortex mixing. The absorption of the upper phase was read at 520 nm using toluene as a blank.

### Cadmium and Mineral Analysis

The dried leaf, fruit, and root tissues were ground in a Wiley mill to pass through a 20-mesh screen, then 0.5 g samples were analyzed for the following macro- and micro-nutrients and toxic element: P, K, Ca, Mg, Fe, Mn, Zn, and Cd. The Cd concentration in the fruit tissue was also determined. The elements were determined by dry ashing at 400°C for 24 h, dissolving the ash in 1:20 HNO_3_, and assaying the solution obtained using an inductively coupled plasma emission spectrophotometer (ICP Iris; Thermo Optek, Milano, Italy; Karla, 1998).

### Metabolomic Analysis

The screening of plant metabolites was carried out on a hybrid quadrupole-time-of-flight mass spectrometer coupled to an UPLC chromatographic system (UPLC/Q-TOF). The instrument was run in the positive scan mode and operated to acquire spectra in the range of 100–1600 m/z in extended dynamic range mode. A 1290 liquid chromatograph system, equipped with a binary pump and a Dual Electro spray JetStream ionization system, then coupled to a G6550 mass spectrometer detector (all from Agilent Technologies, Santa Clara, CA, USA) was used. Reverse phase chromatographic separation was performed using an Agilent ZorbaxEclipse-plus column (75 mm × 2.1 mm i.d., 1.8 μm). The LC mobile phase A consisted of water while mobile phase B was methanol. Formic acid 0.1% (v/v) and ammonium formate (5 mM) were added to both phases. The gradient started with 5% B and increased to 90% B within 30 min, then held for 5 min. The mobile phase temperature was set to 35°C, the injection volume was 3 μl and the flow rate was 220 μl/min. Samples were grinded in liquid nitrogen, then an aliquot (0.5 g) was extracted in 80% methanol with 0.05% HCOOH using an Ultra Turrax (Ika T-25, Staufen, Germany), diluted in 40% methanol and transferred to a vial for analysis by UPLC/Q-TOF.

Raw data gained from the time-of-flight analyzer were processed by the MassHunter Qualitative Analysis B.05 software (from Agilent Technologies) using the naïve “find-by-molecular-feature” algorithm. Unidentified molecular features were subjected to a recursive analysis workflow using Mass Profiler Professional B.12.05 (from Agilent Technologies) for features alignment and filtering after the initial deconvolution. Features that were not present in 80% of replications within a treatment were discarded. Compound identification was based on accurate mass and isotope pattern (accurate spacing and isotopes ratio) and expressed as overall identification score, using the database exported from PlantCyc9.5 (Plant Metabolic Network, http://www.plantcyc.org; released November 2014) as reference.

The peak volume of those compounds identified with a mass accuracy below 5 ppm and above 85/100 as overall identification score, were extracted from the total ions current and exported for statistics and data interpretation.

### RNA Extraction and RT-PCR Analysis

Total RNA was extracted using RNAeasy^®^ Plant Mini Kit (Qiagen, Italy) according to manufacturer’s instructions. The RNA concentration was determined both spectrophotometrically and by densitometric analysis of rRNA fragment following agarose gel electrophoresis. QuantiTect^®^ Reverse Transcription Kit (Qiagen) was used to remove genomic DNA contamination and to synthesize cDNA. Elimination of genomic DNA from cDNA preparation was verified by PCR with primers aligned in different exons for gene EF α1 (elongation factor α1) as described by Expósito-Rodríguez et al. (2008). The quantitative real-time PCR experiments were performed using the iCycler (Bio-Rad, Italy) and using master mix iQTMSYBER Green Supermix (Bio-Rad, Italy), containing the SYBR Green I DNA binding dye. Each reaction was made in triplicate. Primers were used in this study as described by Hartke et al. (2013) and following sequences (sense and antisense, respectively):*LeNRAMP3* F 5′-TGGTTAACTGGATTGTTGGCTGCTGGAC-3′ and *LeNRAMP3* R 5′-ATGAATTCAAAGAAGGGGGATCAGGGCA-3′ for *LeNRAMP3* (Natural Resistance-Associated Macrophage Protein 3); *LeFER* (Ferritin) F 5′-TGGTTAACTCCAACAAGCAAAGGCACGA-3′ and *LeFER* R 5′-ATGAATTCAGAAGCTGCAATGTGTCGCC-3′ for *LeFER*; UBI3 F 5′-TGCAGATCTTCGTGAAAACC-3′ and UBI3 R 5′-AGCGAGCTTAACCTTCTTCT-3′ for UBI3 (Ubiquitin). Total reaction volume was 20 μl that included 10 μl (2X) master mix, 100 ngof cDNA, 0.5 μl (10 μM) of each forward and reverse primers and volume was adjusted with water. The PCR reaction conditions were: one cycle at 50°C for 2 min, 94°C for 15 min, then 40 cycles at 95°C for 15 s, 60°C for 50 s and 72°C for 50 s. Primer specificity was confirmed by nucleotide sequencing (MWG, Germany) of amplicon. The Ct values of target genes (*LeNRAMP3* and *LeFER*) and reference gene (UBI3) were used for further relative expression analysis by using the 2^-ΔΔCT^method (Livak and Schmittgen, 2001). Calculation and statistical analyses were performed by Gene Expression Macro^TM^ Version 1.1 (Bio-Rad, Italy). The qRT-PCR experiments performed for three biological replicas containing pooled samples of leaves and roots of three plants for each treatment.

### Statistical Analysis

All data were statistically analyzed by ANOVA using the SPSS software package (SPSS 10 for Windows, 2001). To separate treatment means within each measured parameter, Duncan’s multiple range test was performed at *P* = 0.05.

Interpretation of metabolomic analysis was carried out using Mass Profiler Professional B.12.05 (from Agilent Technologies). The identified compounds were log2 normalized, filtered (only those having a peak volume above 5000 counts and appearing in 80% of samples in at least one condition, were considered), normalized at 75th percentile and baselined versus the median of each compound in all samples. Statistics and interpretations were performed on this latter dataset: ANOVA analysis (*P* = 0.001, Bonferroni multiple testing correction) and fold-change analysis (cut-off = 5) were combined into volcano plots.

The “find-by-minimal-entities” naïve Bayesian approach in Mass Profiler Professional, aimed to identify those compounds (target size 30 compounds, forward selection algorithm) that better explain differences among treatments, was also carried out.

The third statistical approach was the multivariate Partial Least Square Discriminant Analysis (PLS-DA), a covariance-based prediction model. The score plot for the first latent vector was generated from the model, and those entities having a score higher than 0.1 or lower than -0.1 were considered.

## Results

### Root Mycorrhizal Colonization

Neither hyphae nor vesicle were observed in roots of non-inoculated tomato plants. The rates of mycorrhizal colonization in all inoculated treatments were generally high ranging from 64 to 82% (data not shown). Irrespective of grafting combination (Cd × mycorrhizal interaction, **Table 1**), the percentage of root colonization was higher at 0 μM (76%) than at 25 μM Cd (68%). Moreover, when averaged over Cd concentration (mycorrhizal × grafting interaction, **Table 1**), tomato plants grafted onto Maxifort rootstock had more root colonization (77%) than self-grafted inoculated ones (67%).

**Table 1:** Mean effects of solution cadmium (Cd) concentration, arbuscular mycorrhizal (AM) inoculation, and grafting combination on root mycorrhizal colonization, fruit yield, leaf area, dry weight biomass, and root to shoot ratio of tomato plants.

Treatment	Root AM colonization (%)	Fruit yield (kg plant^-1^)	Dry biomass (g plant^-1^)		Root to Shoot ratio	Leaf area (m^2^ plant^-1^)
			Shoot	Root		
**Cd level (μM)**						
0	38.2	2.82	214.9	25.4	0.122	2.16
25	34.3	2.48	163.6	20.5	0.122	1.83
**AM fungi**						
−AM	0.0	2.70	192.8	22.0	0.120	2.01
+AM	72.5	2.59	185.8	24.1	0.123	1.98
**Grafting combination^**a**^**						
I/I	33.7	2.49	181.8	21.4	0.116	1.90
I/M	38.8	2.81	196.7	24.7	0.128	2.09
**Significance^**b**^**						
Cd	*	***	***	***	NS	***
AM fungi (M)	***	NS	NS	*	NS	NS
Grafting (G)	**	***	**	**	*	***
Cd × M	*	*	**	NS	NS	NS
Cd × G	NS	NS	NS	NS	NS	NS
Cd × M	**	NS	NS	NS	NS	NS
Cd × M × G	NS	NS	NS	NS	NS	NS

*^a^I/I = self-grafted Ikram cultivar; I/M = Ikram cultivar grafted onto Maxifort rootstock.*

^b^NS, Non-significant; *P < 0.05; **P < 0.01; ***P < 0.001.

### Plant Biomass Accumulation, Leaf Area, Leaf Pigments, and Chlorophyll Fluorescence

Irrespective of grafting combination (Cd × mycorrhizal interaction, **Table 1**), the marketable yield and shoot biomass remained unchanged in both +AM and −AM plants at 0 μM Cd (avg. 2.8 kg plant^-1^ and 214.9 g plant^-1^, respectively). At 25 μM Cd the yield and shoot dry weight were significantly higher by 11 and 15%, respectively, in −AM (avg. 2.6 kg plant^-1^ and 174.8 g plant^-1^, respectively) in comparison to those recorded with +AM plants (avg. 2.3 kg plant^-1^ and 152.6 g plant^-1^, respectively). Long-term Cd treatment caused significant decrease in the root dry weight, and leaf area (**Table 1**). The yield, shoot and root biomass, root-to-shoot ratio, and the leaf area were 13, 8, 16, 10, and 10% higher with tomato grafted onto Maxifort than self-grafted plants (**Table 1**).

Chlorophyll *a*, chlorophyll *b*, carotenoid contents, and maximum quantum use efficiency of PSII measured as the F_v_/F_m_ ratio showed significant reduction (by 22, 20, 19, and 3%, respectively) in Cd-exposed plants. Moreover, the chlorophyll *a*, carotenoid contents, and F_v_/F_m_ ratio increased by 8, 13, and 2%, respectively, when tomato plants were grafted onto Maxifort (**Table 2**).

**Table 2:** Mean effects of solution Cd concentration, AM inoculation, and grafting combination on pigment content and maximum quantum use efficiency of PSII (F_v_/F_m_) in tomato leaves.

Treatment	Pigments (μg g^-1^fw)			Fv/Fm
	Chlorophyll a	Chlorophyll b	Carotenoids	
**Cd level (μM)**				
0	490.3	208.2	183.7	0.833
25	383.9	166.5	149.4	0.810
**AM fungi**				
−AM	422.7	180.5	164.3	0.821
+AM	451.5	194.1	168.8	0.819
**Grafting combination^a^**				
I/I	420.6	184.0	156.4	0.815
I/M	453.6	190.7	176.7	0.824
**Significance^b^**				
Cd	***	***	***	***
AM fungi (M)	NS	NS	NS	NS
Grafting (G)	*	NS	**	*
Cd × M	NS	NS	NS	NS
Cd × G	NS	NS	NS	NS
Cd × M	NS	NS	NS	NS
Cd × M × G	NS	NS	NS	NS

*^a^I/I = self-grafted Ikram cultivar; I/M = Ikram cultivar grafted onto Maxifort rootstock.*

^b^NS, Non-significant; *P < 0.05; **P < 0.01; ***P < 0.001.

### Oxidative Damage

**FIGURE 1:**
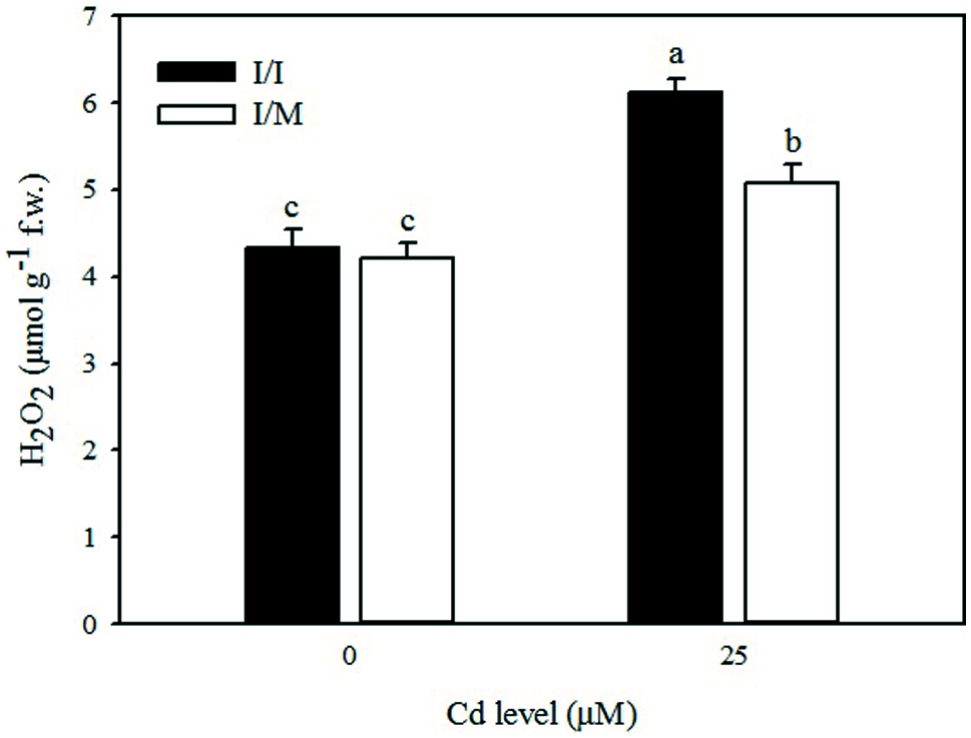
**Effects of grafting combination (I/I = self-grafted Ikram cultivar; I/M = Ikram cultivar grafted onto Maxifort rootstock) and solution cadmium (Cd) concentration on hydrogen peroxide (H_2_O_2_) content in leaves of tomatoes.** Different letters indicate significant differences according to the Duncan’s multi-range test (*p* ≤ 0.05).

As shown in **Figure 1**, the leaves of tomato cv. Ikram grafted onto Maxifort displayed lower Cd-induced hydrogen peroxide (H_2_O_2_) accumulation (-17%) than those of self-grafted plants, whereas no significant difference of H_2_O_2_ production was recorded between the two grafting combinations under control conditions (0 μM Cd). The Cd treatment produced a consistent increase in the level of lipid peroxidation in leaves determined by measuring the amount of MDA and also an increase in EL. The MDA content and EL recorded on plants receiving 25 μM Cd was significantly higher by 28 and 9%, respectively, in comparison to tomato plants treated with 0 μM Cd (**Table 3**). The MDA content and EL were also affected by grafting combinations with the maximum values recorded in self-grafted than in Ikram/Maxifort combination (**Table 3**).

**Table 3:** Mean effects of solution Cd concentration, AM inoculation, and grafting combination on hydrogen peroxide (H_2_O_2_), malondialdehyde (MDA), and electrolyte leakage in tomato leaves.

Treatment	H_2_O_2_	MDA	Electrolyte
	(μmol g^-1^f.w.)	(nmol g^-1^f.w.)	leakage (%)
**Cd level (μM)**			
0	4.28	4.65	68.18
25	5.59	5.95	74.10
**AM fungi**			
−AM	4.84	5.22	70.71
+AM	5.03	5.39	71.57
**Grafting combination^a^**			
I/I	5.23	5.40	73.23
I/M	4.64	5.21	69.05
**Significance^b^**			
Cd	***	***	**
AM fungi (M)	NS	NS	NS
Grafting (G)	*	*	*
Cd × M	NS	NS	NS
Cd × G	*	NS	NS
Cd × M	NS	NS	NS
Cd × M × G	NS	NS	NS

*^a^I/I = self-grafted Ikram cultivar; I/M = Ikram cultivar grafted onto Maxifort rootstock.*

^b^NS, Non-significant; *P < 0.05; **P < 0.01; ***P < 0.001.

### Effects on Antioxidative Enzymes

**FIGURE 2:**
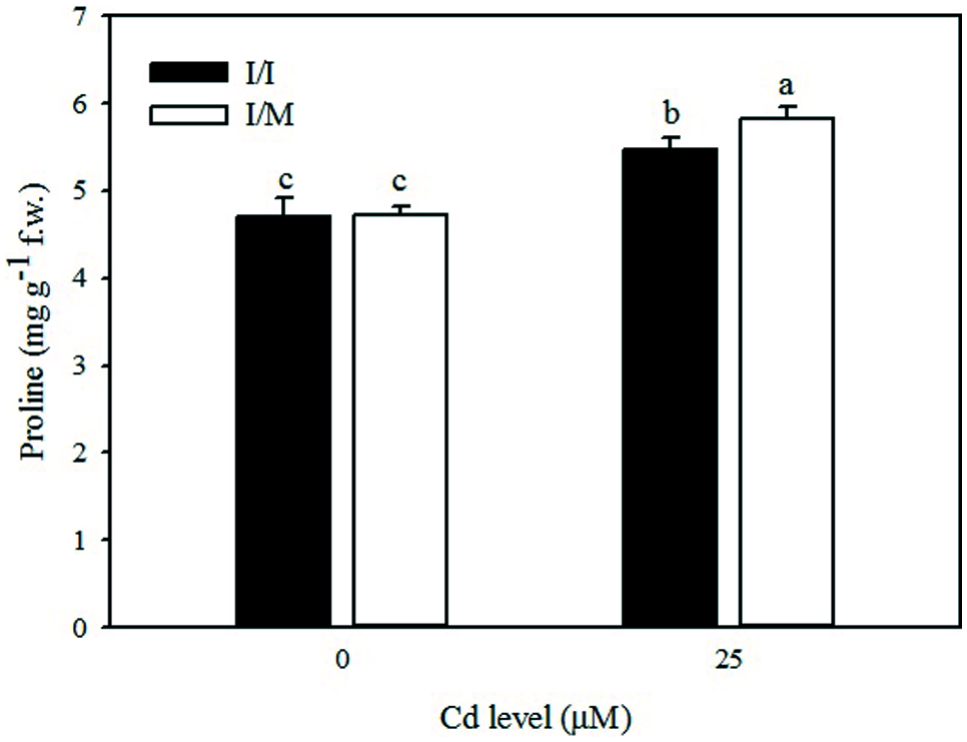
**Effects of grafting combinations (I/I = self-grafted Ikram cultivar; I/M = Ikram cultivar grafted onto Maxifort rootstock) and solution Cd concentration on proline content in leaves of tomatoes.** Different letters indicate significant differences according to the Duncan’s multi-range test (*p* ≤ 0.05).

Stress response of grafted and self-grafted plants with and without AM exposed to moderate Cd-stress was assessed by analyzing changes in capacity of several antioxidative enzymes on primary leaves (**Table 4**). Irrespective of grafting combination (Cd × mycorrhizal interaction), CAT activity in leaves remained unchanged in both +AM and −AM plants at 25 μM Cd (622.1 mmol mg^-1^protein min^-1^), whereas the CAT activity was higher by 44% in −AM (570.1 mmol mg^-1^protein min^-1^) in comparison to the one recorded with +AM plants (396.2 mmol mg^-1^protein min^-1^). APX and GPX activities in the leaves of moderate Cd-exposed tomato plants were higher by 9% and lower by 15%, respectively, than in the control (**Table 4**). Among the grafting combinations, the lowest APX and GPX activities were recorded in the Ikram/Ikram combination. The proline content in leaves of tomato cv. Ikram grafted onto Maxifort was higher by 7% than the one recorded on self-grafted plants under Cd-stress conditions, whereas no difference in proline was observed between the two grafting combinations under control treatment (**Figure 2**).

**Table 4:** Mean effects of solution Cd concentration, AM inoculation and grafting combination on antioxidant enzymes catalase (CAT), ascorbate peroxidase (APX), guaiacol peroxidase (GPX), and proline contents in tomato leaves.

Treatment	Antioxidant enzymes (mmol min^-1^ mg^-1^ protein)			Proline (mg g^-1^fw)
	CAT	APX	GPX	
**Cd level (μM)**
	483.0	11.6	2.61	4.72
25	621.9	12.7	2.17	5.65
**AM fungi**
−AM	601.6	12.6	2.47	5.20
+AM	503.4	11.7	2.31	5.17
**Grafting combination^a^**
I/I	51.69	11.6	2.24	5.09
I/M	58.80	12.8	2.55	5.28
**Significance^b^**
Cd	***	*	***	***
AM fungi (M)	***	*	NS	NS
Grafting (G)	**	**	*	NS
Cd × M	**	NS	NS	NS
Cd × G	NS	NS	NS	*
Cd × M	NS	NS	*	NS
Cd × M × G	NS	NS	NS	NS

*^a^I/I = self-grafted Ikram cultivar; I/M = Ikram cultivar grafted onto Maxifort rootstock.*

^b^NS, Non-significant; *P < 0.05; **P < 0.01; ***P < 0.001.

### Metabolic Profiling of Leaves

The combination of a comprehensive database like Plant Cyc together with proper data handling (mass and retention time alignment, filtering by frequency and following recursive analysis) was effective in removing false positives while ensuring a thorough dataset. Indeed, starting from a total of 2242 compounds detected, recursive analysis and the following filters in Mass Profiler Professional dramatically reduced the number of compounds in the dataset and provided better interpretations.

**FIGURE 3:**
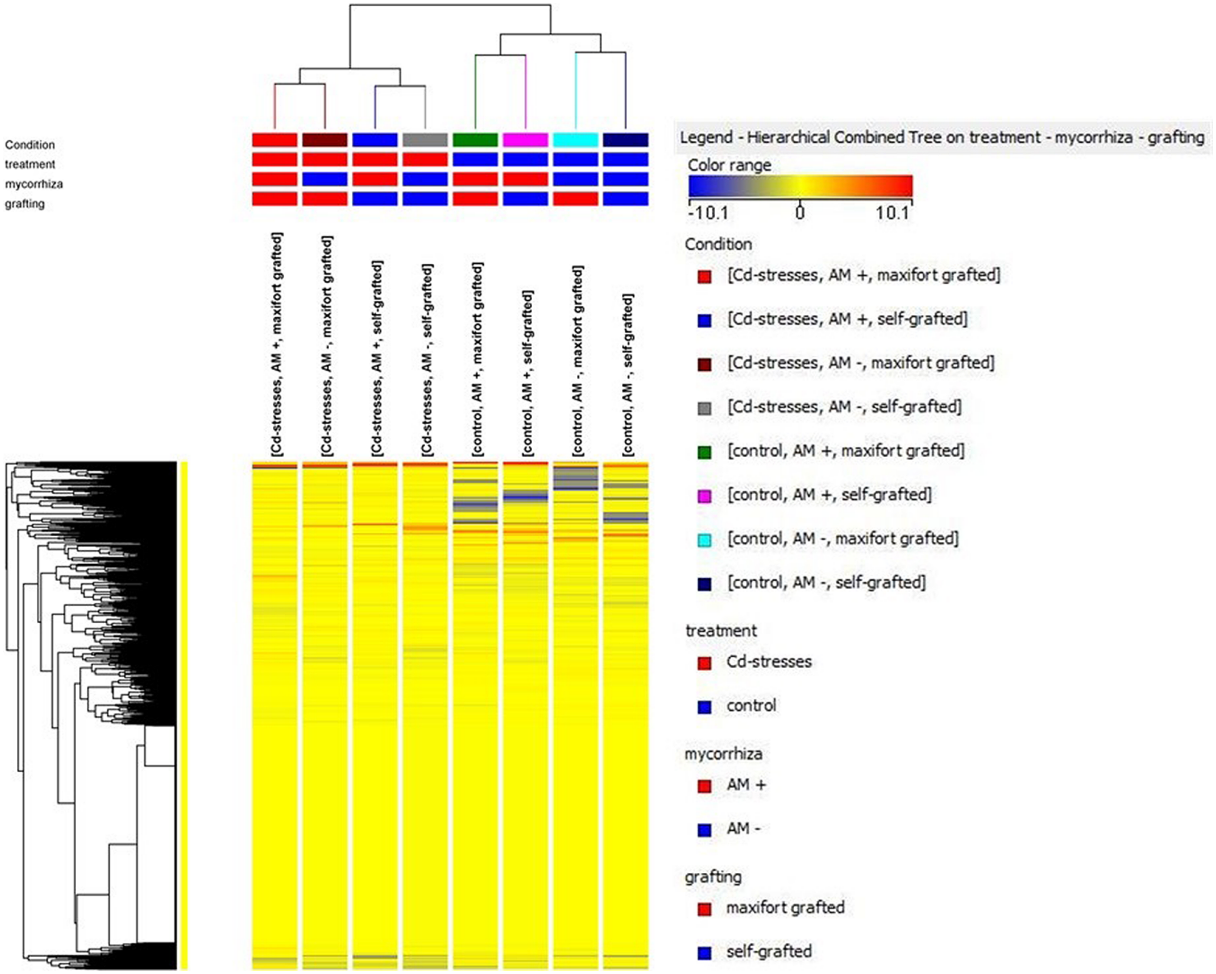
**Unsupervised hierarchical cluster analysis of metabolite contents in all treatments**.

**FIGURE 4:**
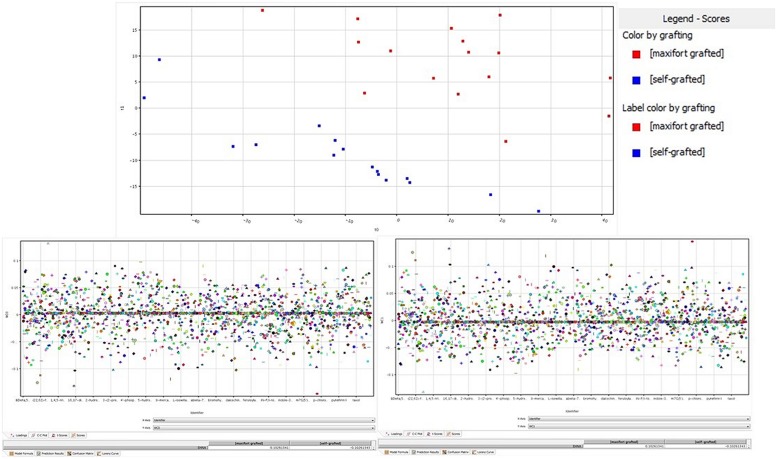
**Partial least squares discriminant analysis (PLS-DA) identification of metabolites being significantly altered by grafting. (Upper)** panel is the hyperspace plot from the PLS-DA model, while lower panes are the class prediction loadings on first **(Left)** and second **(Right)** hyperspace components.

The unsupervised cluster analysis (**Figure 3**) performed on the dataset gave good clustering, considering that the treatments were properly grouped according to the stress applied (Cd-stressed versus control) and then to grafting combination and mycorrhizal inoculation. The effect of the latter was secondary to grafting. Furthermore, the output of PLS-DA multivariate analysis carried out on grafting combinations (samples position in the model hyperspace, together with score plot for first and second later vectors) is given in **Figure 4**. The class prediction models gave good accuracies for both grafting and mycorrhization (overall accuracy was 96.9 and 100%, respectively). The compounds selected from Volcano analysis (using a fold-change cut off = 5 and a *p*-value of 0.001), PLS-DA scores, and naïve Bayesian analysis were summarized in **Tables 5** and **6**, grouped in classes according to their biological role.

**Table 5:** Variation of differential metabolites in tomato leaves under Cd stress (25 μM) and absence of mycorrhization, in response to grafting combination (Ikram/Maxifort vs. Ikram/Ikram).

Metabolites	Variation
***Phenolics***	
(+)-sesaminol 2-*O*-β-D-gentiobioside	+
Quercetin-3,3′-bissulfate	+
Ponciretin	−
***Alkaloids***	
Harmalol	+
(*S*)-norcoclaurine	−
***Glucosinolates***	
Indole-3-acetyl-aspartate-*N*-β-D-glucose	+
8-methylthiooctyldesulfoglucosinolate	−
2-(6′-methylthio) hexylmalate	−
***Lipoperoxidation productsand membrane lipids***	
6-cis-3-oxo-tridecenoyl-CoA	−
22-hydroxydocosanoate	−
1-18:3-2-16:2-monogalactosyldiacylglycerol	+
Heptanal	−
***Hormones***	
Indole-3-acetyl-phenylalanine	−
***Hydroxycinnamic acids amides***	
Dihydroxyferuloyl-sinapoylspermidine	−
Feruloylserotonin	−
***Fructaninulins***	
1,1-kestotetraose	+
Kestotriose	+
**Others**	
Anthranilate	+
Dopaxanthinquinone	−
Ubiquinone-8	−
*D*-galactosylononitol	+
*N^1^,N^2^*-formyl-5-methoxykynuramine	+
(1*E*,6*E*)-1-(4-hydroxyphenyl)-7-phenylhepta-1, 6-diene-3,5-dione	+
*L*-asparagine	+
Precorrin-2	−
PC2 (γ-Glu-Cys-γ-Glu-Cys-β-Ala)	+

*Data are gained from Volcano analysis (alpha 0.01, fold-change = 5) and Partial least squares discriminant analysis (PLS-DA) multivariate analysis.*

**Table 6:** Differential metabolites in tomato leaves of self-grafted plants (Ikram/Ikram) under Cd stress (25 uM) in response to AM root inoculation (+AM vs. −AM).

Metabolites	Variation
***Phenolics***	
Apigenin 7-*O*-β-*D*-glucoside	−
7-*O*-β-*D*-glucosyl-7-hydroxyflavone	−
Quercetin-3,3′-bissulfate	+
***Alkaloids***	
Harmaline	−
Harmalol	+
***Glucosinolates***	
4-methoxy-3-indolylmethylisothiocyanate	+
Indole-3-acetyl-aspartate-*N*-β-*D*-glucose	+
Tetrahomomethionine	+
6-hydroxy-indole-3-acetyl-phenylalanine	−
***Lipoperoxidation products and membrane lipids***	
1-18:3-2-16:2-monogalactosyldiacylglycerol	+
1-dodecanol	−
1-18:2-2-16:0-monogalactosyldiacylglycerol	−
***Hormones***	
(-)-medicarpin	−
(-)-sativan	−
Indole-3-acetyl-phenylalanine	−
***Hydroxycinnamic acids amides***	
Dihydroxyferuloyl-sinapoylspermidine	−
Feruloylserotonin	+
***Phytochelatins related compounds***	
acryloyl-CoA	−
γ-glutamyl-ethylamide	−
**Others**	
Anthranilate	+
*N*-*acetyl*-*L*-*glutamate*	+
α-ethyl-*L*-glutamate	−
Dopaxanthinquinone	−
Ubiquinone-8	−
*N*-δ-(phosphonoacetyl)-L-ornithine	−
D-galactosylononitol	+
Precorrin-2	−
1,7,8-trihydroxy-6-methoxy-2-methylanthraquinone	+
1,7-dihydroxy-5,6,8-trimethoxyanthraquinone	−
*N,N*-dihydroxy-L-tryptophan	+
4-methylumbelliferone 6′-*O*-malonylglucoside	−
4-coumaroylhexanoylmethane	−

*Data are gained from Volcano analysis (alpha 0.01, fold-change = 5) and PLS-DA multivariate analysis.*

The main classes of compounds identified were ascribed to hormones (indole acetic acid derivatives, the phytoalexins medicarpin and sativan, some inactive forms of gibberellins and a brassinosteroid, glucosinolates, indole and benzylisoquinoline alkaloids, hydroxycinnamic acids amides, and several products of lipoperoxidation (oxo- and hydroxyl-derivatives of lipids). Phenolic compounds (flavonoids and lignans), together with terpenes, were also altered in response to the treatments. Some additional compounds, not directly ascribable to the previous classes, were also pointed out. These latter included other metabolites, such as glutamate derivatives (*N*-acetyl-glutamate, *N,N*-dihydroxyglutamate, α-ethyl-glutamate), phytochelatin PC2, fructan inulins, a melatonin intermediate (*N^1^*-acetyl-*N^2^*-formyl-5-methoxykynuramine), and quinones/quinols (dopaxanthinquinone, ubiquinone-8, and menaquinol-7).

Interestingly, the outcome of the multivariate PLS-DA provided a ranked list of metabolite features in good agreement with the previous Volcano analyses. Hence, a few additional compounds still belonging to the functional classes reported above were added by this approach. Finally, a few compounds were pointed out through the naïve Bayesian analysis, mainly regarding the contribution of grafting, namely the methyl-THF (hence a methyl-donor group) and the two forms of ascorbate.

### Cd Accumulation in Tomato Tissues

**FIGURE 5:**
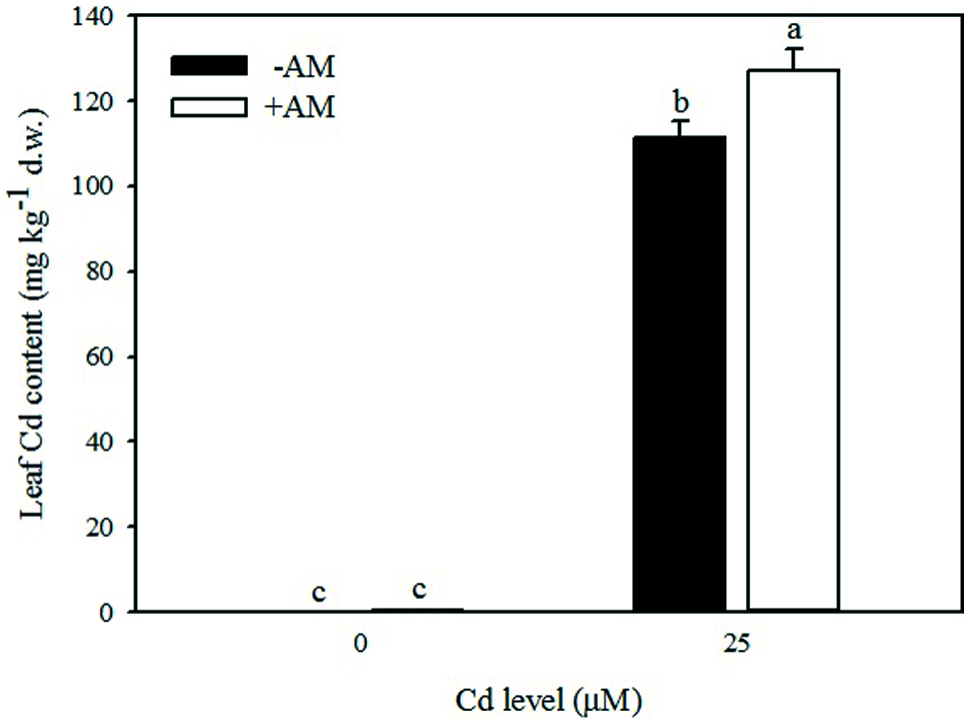
**Effects of arbuscular mycorrhizal (AM) inoculation and solution Cd concentration on Cd content in leaves of tomatoes.** Different letters indicate significant differences according to the Duncan’s multi-range test (*p* ≤ 0.05).

In the absence of added Cd in the nutrient solution, very low concentrations of Cd accumulated in leaves (avg. 0.35 mg kg^-1^, **Table 7**), roots (avg. 0.38 mg kg^-1^, data not shown), and fruits (0.08 mg kg^-1^, data not shown). Addition of Cd in the nutrient solution greatly increased the Cd concentration in roots (475.1 mg kg^-1^, data not shown), leaves (119.1 mg kg^-1^, **Table 7**), and fruits (5.1 mg kg^-1^, data not shown), confirming that roots are the primary site of Cd accumulation. In the aerial tissues, the Cd concentration changed with AM fungi inoculation. At 25 μM Cd, the Cd concentration was higher by 14% in leaves of +AM than in leaves of −AM plants (**Figure 5**).

**Table 7:** Mean effects of solution Cd concentration, AM inoculation, grafting combination on major, and trace elements in tomato leaves.

Treatment	Major elements (g kg^-1^ of dw)					Trace elements (mg kg^-1^dw)			
	N	P	K	Ca	Mg	Fe	Mn	Zn	Cd
**Cd level (μM)**						
0	26.4	3.38	24.1	37.2	7.54	74.5	300.3	33.9	0.35
25	25.3	3.28	23.9	32.2	6.55	38.0	265.1	23.7	119.14
**AM fungi**						
−AM	25.7	3.20	23.2	33.7	6.82	60.3	280.6	27.1	55.73
+AM	26.1	3.46	24.9	35.7	7.27	52.1	284.8	30.5	63.75
**Grafting combination^a^**						
I/I	26.1	3.20	22.0	31.9	7.01	53.6	251.6	26.7	58.06
I/M	25.6	3.46	26.1	37.4	7.08	58.8	313.8	30.9	61.42
**Significance^b^**						
Cd	NS	NS	**	**	***	***	***	***	***
AM fungi (M)	NS	*	NS	NS	*	**	NS	**	***
Grafting (G)	NS	*	***	***	NS	*	***	**	NS
Cd × M	NS	NS	NS	*	*	**	NS	NS	***
Cd × G	NS	NS	NS	NS	NS	NS	NS	*	NS
Cd × M	**	NS	NS	NS	NS	NS	NS	NS	NS
Cd × M × G	***	*	NS	NS	NS	*	NS	*	NS

*^a^I/I = self-grafted Ikram cultivar; I/M = Ikram cultivar grafted onto Maxifort rootstock.*

^b^NS, Non-significant; *P < 0.05; **P < 0.01; ***P < 0.001.

### Mineral Concentrations in Leaf Tissue

Increasing the Cd concentration in the nutrient solution from 0 to 25 μM, decreased the leaf mineral composition in particular K, Ca, Mg, Fe, Mn, and Zn by 7, 13, 13, 49, 12, and 30%, respectively (**Table 7**). The effect of AM inoculation on mineral concentration in leaf tissues was less pronounced. Except for Fe concentration, the better nutritional status (higher P, Mg, and Zn) was recorded with +AM than in −AM plants (**Table 5**). Macro and micronutrient concentrations were also affected by grafting combination, with the highest values of P, K, Ca, Fe, Mn, and Zn observed in shoot tissues of Ikram grafted onto Maxifort rootstock (**Table 7**).

The concentration of certain nutrients (Fe, Zn, and Mg) in the root tissue was significantly increased with 25 μM Cd (avg. 375.6 mg kg^-1^, 96.4 mg kg^-1^, and 2.13 g kg^-1^, respectively) compared to control treatment (avg. 255.4 mg kg^-1^, 77.9 mg kg^-1^, and 1.76 g kg^-1^, respectively), whereas Mn decreased with Cd treatment (from 110.8 to 98.0 mg kg^-1^). Finally, Fe, Mn, and Cu concentrations were significantly higher in Ikram/Maxifort (avg. 330.7, 108.7, 80.1 mg kg^-1^, respectively) compared to Ikram/Ikram combination (avg. 300.2, 100.1, 60.3 mg kg^-1^, respectively).

### Gene Expression

The relative gene expression analysis of two important metal transporters *LeFER* (root specific transporter) and *LeNRAMP3* genes (a wide range metal transporter) was performed. Under Cd-stress, the *LeFER* and *LeNRAMP3* genes were up-regulated in non-inoculated roots (about twofold) of both Ikram/Ikram, and Ikram/Maxifort combinations. This seems to be related to root Cd concentrations, which was similarly increased in both grafting combinations (**Table 8**). When *LeNRAMP3* was analyzed in leaves, it showed up-regulation only in Ikram/Maxifort combination, with and without AM inoculation (twofold and threefold, respectively), whereas a down-regulation trend was observed in self-grafted plants (Ikram/Ikram) with and without AM inoculation (**Table 8**). Moreover, at 25 μM Cd, the *LeFER* and *LeNRAMP3* genes showed down-regulation in mycorrized roots of both grafting combinations (**Table 8**).

**Table 8:** Relative gene expression of *LeFER* and *LeNRAMP3* gene in self grafted (I/I) and Maxifort rootstock grafted (I/M) plants with (+AM) or without (−AM) mycorrhizal inoculation.

Grafting combination	Relative gene expression					


	*LeFER*		*LeNRAMP3*			
	Root		Root		Leaf	
	−AM	+AM	−AM	+AM	−AM	+AM
I/M	1.77 ± 0.2	0.61 ± 0.35	2.05 ± 0.69	0.72 ± 0.19	2.70^a^ ± 0.2	2.01^a^ ± 0.32
I/I	1.78 ± 0.2	0.34 ± 0.17	1.84 ± 0.43	0.63 ± 0.31	0.46 ± 0.2	0.59 ± 0.30

*qRT-PCR analysis was performed in root and leaf tissues following the Cd treatment and mycorrhization in self grafted and Maxifort rootstock grafted plants. Quantification of gene expression was performed using the 2^-ΔΔCT^ method (Livak and Schmittgen, 2001). Values are means of three technical replicates and two biological replicates.*

*^a^Means that significantly differed from the calibrator by student’s *t*-test (*p* < 0.05).*

## Discussion

Plants respond to elevated Cd concentrations in water or soil by decreased yield and crop growth parameters due to Cd-induced inhibition of physiological and metabolomic processes and/or impairment of root activity, nutrient uptake, and accumulation (López-Millán et al., 2009; Djebali et al., 2010; Sharma et al., 2010). However, the response of crops to Cd-stress conditions during the growing cycle may vary in relation to several interacting variables such as species, the phenological stage, the cultural environment, and the magnitude of stress, i.e., time of exposure and Cd concentration; (Lux et al., 2011 and references cited therein). In the current experiment, significant decrease in root colonization and agronomical responses (**Table 1**) in Cd-treated tomato plants was observed; and that effect varied in relation to AM fungi inoculation and grafting combination. The expected positive effect of mycorrhizal fungus colonization on plant growth and productivity was not evident under moderate Cd-stress conditions. At 25 μM Cd, the reduction of yield, shoot, and leaf area production was more pronounced with AM inoculation. This significant reduction in plant performance was attributed to the higher accumulation of Cd in shoot tissues (63.7 vs. 55.7 mg kg^-1^, for +AM and −AM, respectively), which is likely the result of active substrate-Cd transport to the roots via extraradical hyphal network (Burleigh et al., 2003; González-Guerrero et al., 2005). These results are consistent with the findings of Prasad et al. (2011), who reported that AM fungal inoculation with *R. irregularis* decreased the shoot and root yield of sweet basil at low dose of Cd (25 mg kg^-1^), whereas at elevated levels (50 mg kg^-1^) of Cd in soil, *R. irregularis* inoculation showed an opposite behavior. In a recent meta-analysis study on the dynamic roles of AM symbiosis in heavy metal phytoremediation, Audet and Charest (2007) demonstrated a transition role of the AM shifting from ‘enhanced uptake’ at low soil heavy metal levels, to ‘metal binding’ at high soil heavy metal levels. Contrarily to AM symbiosis, the negative effect of moderate Cd on yield, shoot, and root development was clearly attenuated, when tomato cv. ‘Ikram’ was grafted onto the vigorous rootstock Maxifort. Cd tolerance of tomato plants grafted onto Maxifort may be due to the better uptake and translocation of macro and microelements in particular P, K, Ca, Fe, Mn, and Zn to the leaves. The improved nutritional status of vegetables grafted onto vigorous rootstocks under adverse chemical soil conditions may be associated to the higher root-to-shoot ratio and also to the greater surface area necessary for absorption of water and nutrients (Colla et al., 2010; Savvas et al., 2010).

Cadmium stress can cause damage at physiological level by interfering with several aspects of plant biochemistry including pigment synthesis and photosynthesis (Djebali et al., 2005; Hèdiji et al., 2010). In the current experiment, Cd-induced degradation of leaf pigments in particular chlorophyll and carotenoids, as well as inhibition of their biosynthesis (Sandalio et al., 2001; Boswell et al., 2002), was probably a consequence of Cd induced nutrient deficiency (i.e., Fe, Mg; Vazquez et al., 1987; John et al., 2009). Excess Cd concentrations also create disturbance in electron transport rates of the PSII, therefore generating ROS (Djebali et al., 2005; Herbette et al., 2006). Furthermore, the reduction in chlorophyll is a direct consequence of reduced carotenoids content, the latter is known to protect chlorophyll and other macromolecules by quenching excited triplet state of chlorophyll to avoid generation of free radicals (Bartley and Scolnik, 1995; Hèdiji et al., 2010). The negative impact of Cd on leaf pigments and F_v_/F_m_ratio was clearly mitigated when Ikram/Maxifort combination was used (**Table 2**). In self-grafted plants, the higher plant biomass accumulation and yield in Ikram/Maxifort seem to be related to the capacity of maintaining higher photochemical activity in response to Cd-stress. Similar results were observed on grafted tomato and cucumber plants, which were able to delay photo-inhibition under salt stress in comparison to ungrafted plants (He et al., 2009; Colla et al., 2013).

Furthermore, elevated Cd concentration in nutrient solution induced oxidative stress at cellular level as evidenced by enhanced hydrogen peroxide (H_2_O_2_) generation, ion leakage, and lipid peroxidation (**Table 3**; Djebali et al., 2005; Sharma and Dubey, 2005; Smeets et al., 2005; Rodriguez-Serrano et al., 2006). The Ikram/Maxifort combination was more tolerant to Cd stress than the self-grafted plants; this was determined based on lowest values of ROS accumulation and MDA (a lipid peroxidation product). Our results also demonstrated that plants grafted onto Maxifort reduced the amount of ion leakage in Cd stressed tomato thus facilitating the maintenance of membrane functions (i.e., semi permeability). Calcium increases structural stability of cell membrane because of electrostatic interactions with membrane phospholipids and proteins and of its role as fundamental component of the cell wall (Borer et al., 2005). Cd applications in the nutrient solution reduced root calcium uptake leading to a reduction of cell membrane stability of leaf tissues; however, Ikram/Maxifort combination was able to mitigate the detrimental effect of Cd on membrane stability by improving the Ca uptake in leaf tissues of tomato plants.

It is well established that under stress conditions, multiple antioxidant enzymes are expected to play a crucial role in the scavenging of ROS and thus protect cells from oxidative damage (Xu et al., 2012). Antioxidant enzymes, such as CAT, APX, and GPX, responded differently under various Cd conditions. The activities of CAT and APX, the major enzymes responsible for H_2_O_2_ degradation (Vanacker et al., 1998) increased at 25 μM Cd whereas an opposite trend was observed for GPX (**Table 4**). The activities of CAT and APX were significantly higher with −AM in comparison to +AM plants. The Ikram/Maxifort combination induced higher antioxidant enzymes, which suggested that the use of Maxifort rootstock in tomato has a high ROS scavenging activity. This result may explain the lower H_2_O_2_ concentrations that were observed in the presence of Cd (**Figure 1**). Similar results were observed in grafted plants under thermal and salt stress (He et al., 2009; Colla et al., 2013) where the antioxidant enzymes activity was higher in rootstock-grafted plants than in self-grafted plants.

Another system of protection against Cd toxicity includes the synthesis of osmolytes, such as proline, which contribute to stabilization of protein molecules and membranes (Matysik et al., 2002; Yılmaz and Parlak, 2011). In our study, the higher accumulation of proline in Ikram/Maxifort combination supports the observed higher Cd tolerance in grafted than self-grafted plants (**Table 4**).

The results gained from the statistics, carried out on the metabolomic dataset, were consistent with previous assessments and pointed out secondary metabolites that are known to be related to abiotic stress. Glucosinolate biosynthesis was stimulated by mycorrhiza, being up-accumulated in this treatment, while phenolics were up-accumulated in Maxifort-grafted plants. As expected, the whole phytohormone network was imbalanced by both root colonization and grafting in a complex way probably related to hormones cross-talking. Although a clear role of hormone changes could not be identified, the involvement of our differential hormones is consistent with previous literature (Choudhary et al., 2012; Khan et al., 2012).

As an overall consideration, several metabolites can be linked to oxidative stress. In particular, products from lipid peroxidation are reported to be down-accumulated in Maxifort-grafted plants confirming the role of grafting in supporting the quenching of the oxidative stress generated by Cd exposure. Consistently, diacylglycerols and diacylglycerol glycosides were up-accumulated in this treatment indicating a possible preservation of membrane lipids. The reduction in flavonoids in Maxifort grafted plants, indicate a less stringent need to cope with ROS and oxidative stress, therefore enforcing the previous assumption. Some other compounds can be related to the improved ability of Maxifort-grafted plants for coping with Cd stress, like the case of phytochelatin PC2 (γ-Glu-Cys-γ-Glu-Cys-β-Ala) or the fructan inulins kestotriose and 1,1-kestotetraose.

Other abiotic stress related compounds included some alkaloids, and mainly indole and benzylisoquinoline alkaloids, and the melatonin derivative *N^1^*-acetyl-*N^2^*-formyl-5-methoxykynuramine. All these latter compounds were up-accumulated in Maxifort-grafted plants.

The increased accumulation of melatonin is also supported by literature. The most frequently mentioned functions of melatonin are related to abiotic stresses including chemical stresses. Environmental stress can increase the level of endogenous melatonin in plants, and this compound was demonstrated to alleviate the stress (Zhang et al., 2015). Experiments in tomato, specifically indicated the role of melatonin in coping plant stress and oxidative stress in particular (Arnao and Hernández-Ruiz, 2013). Monoterpene indole alkaloids are also reported to be active against oxidative stress (Matsuura et al., 2014), and therefore, they probably acted in this direction in our experiments. The role of *N*-acetyl-L-glutamate in contrasting abiotic stress, though less obvious, has been previously reported in literature (Kalamaki et al., 2009), whereas the role of phytochelatins and inulin in Cd stress tolerance is more obvious. Hydroxycinnamic acid amides were found to be related to both mycorrhiza and abiotic stress (Jin et al., 2003; Choudhary et al., 2012) thus supporting their presence among differential metabolites.

The naïve Bayesian analysis, mainly regarding the contribution of grafting, pointed out the involvement of methyl-THF (hence a methyl-donor group) and the two forms of ascorbate. Although these compounds were not selected by fold-change analysis, they resulted as differential through this complementary approach.

Cadmium may interfere with nutrient uptake by affecting the permeability of plasma membranes and thereby nutrient composition in plant tissues (Dong et al., 2006; Bertoli et al., 2012). A significant reduction of essential elements – in particular those with the same valence as Cd, such as, Ca, Mg, Fe, Mn, and Zn -was found at 25 μM Cd (**Table 7**). A decrease in the contents of such nutrients could be attributed to the competitive inhibition between these cations and Cd (Bertoli et al., 2012). At 25 μM, the Cd concentration in roots was fivefold higher than in the aerial part suggesting that Cd transport to the xylem was restricted in most plants (Lux et al., 2011 and references cited therein). The amount of Fe in roots increased with increasing contents of Cd, suggesting synergetic effect of Cd by stimulating Fe absorption (López-Millán et al., 2009; Bertoli et al., 2012), whereas an antagonistic effect was observed for Mn in line with the findings of Dong et al. (2006) and Wu et al. (2007b). It was also noteworthy that fruit developing on 25 μM-treated plants accumulated low amounts of Cd (avg. 5.1 mg kg^-1^). This is consistent with previous results showing limited accumulation in tomato fruits grown on contaminated soils (Gupta et al., 2008) and in hydroponic conditions at 20 μM Cd (Hèdiji et al., 2010).

Data of this study also showed that Cd concentration in tomato leaves was higher in inoculated than non-inoculated plants grown under moderate Cd-stress (**Table 7**). This indicated that Cd was not retained in intra-radical AM fungi or compartmentalized in the root cell vacuoles leading to translocation of Cd in the aerial parts, with detrimental effect on plant performance. Our results are in agreement with the meta-analysis of Audet and Charest (2007) who demonstrated that heavy metal uptake is enhanced at low to intermediate soil heavy metal concentrations.

Grafting experiments have suggested that root characteristics (i.e., length, density, surface area) of several rootstocks act as a barrier in restricting Cd (exclusion mechanism) to shoots in eggplant and cucumber (Arao et al., 2008; Mori et al., 2009; Yamaguchi et al., 2011; Savvas et al., 2013). This was not the case in the current study since similar Cd concentration was recorded in both grafting combinations (**Table 7**). Nevertheless, plant tolerance to heavy metals can vary with specific metal and plant species in question and more than one mechanism can be involved in mitigating the toxic effect (Hall, 2002). For instance, it is reported that plant tolerance strategy can not completely rely on exclusion mechanism as in the case of Cd due to possible exclusion of certain essential cations sharing similar transmembrane for uptake (Hall, 2002). The higher crop performance of Ikram/Maxifort in comparison to Ikram/Ikram combination was due to the improved nutritional status (higher P, K, Ca, Fe, Mn, and Zn, **Table 7**). The higher root to shoot ratio and the higher selective root uptake of elements in Ikram/Maxifort combination than in self-grafted plants can explain the better leaf nutritional status.

Finally, the plant response to Cd stress was also evaluated at molecular level by gene expression analysis (**Table 8**). Under Cd-stress, *LeFER* and *LeNRAMP3* genes were up-regulated in roots in both grafting combinations – as also observed by Hartke et al. (2013). However, the up-regulation of *LeNRAMP3* gene only occurred in leaf of Ikram/Maxifort combination that was related to the better nutritional status (higher Fe, Mn, and Zn; **Table 7**). Previous studies have revealed similar functions of some NRAMP genes, e.g., *OsNRAMP3* for Mn (Yang et al., 2013) and *OsNRAMP5* for Fe, Mn, and Zn (Ishimaru et al., 2012a,b and Sasaki et al., 2012) in rice, and *LeNRAMP3* for Fe in tomato (Bereczky et al., 2003). The down-regulation of both the genes observed in +AM roots could be attributed to dilutive effects or transcriptional and proteome changes (Ouziad et al., 2005; Aloui et al., 2011).

## Conclusion

Growth and yield of tomato was restricted by the application of moderate levels of Cd, which could occur in vegetable production systems. The deleterious effects of Cd on plant performance reflect changes in physiological, biochemical, and metabolic processes. AM inoculation was not able to alleviate the detrimental effect of Cd on growth and productivity because Cd could not be retained in intra-radical AM fungi or compartmentalized in the root cell vacuoles, leading to translocation of Cd in the aerial parts. Our study showed that grafting tomato, involving vigorous rootstock such as Maxifort, could effectively mitigate adverse effects of Cd stress by improving plant nutritional status, photosynthetic pigments, photochemical activity of PSII, increase the capacity of antioxidant enzymes (CAT, APX), proline and metabolites linked to oxidative stress or clearly related to Cd tolerance (i.e., phytochelatin, fructans, and inulins). Hence, the contribution of Maxifort rootstock minimized the level of Cd-induced oxidative injury by decreasing the level of hydrogen peroxide, lipid peroxidation, and electrolyte leakage in tomato leaves, thus promoting the performance of tomato plants.

## Conflict of Interest Statement

The authors declare that the research was conducted in the absence of any commercial or financial relationships that could be construed as a potential conflict of interest.
